# Tuning up STAT1

**DOI:** 10.1371/journal.pbio.1002201

**Published:** 2015-07-21

**Authors:** Caitlin Sedwick

**Affiliations:** Freelance Science Writer, San Diego, California, United States of America

## Abstract

STAT1 is a key regulator of diverse immune responses, but the basis of this diversity is poorly understood; a new study uses a parasite's ability to block STAT1 to identify the host protein TLX as a modulator of STAT1 activity in the brain. Read the accompanying Research Article.

The transcription factor signal transducer and activator of transcription-1 (STAT1) is a linchpin of immunity. In the presence of the inflammatory cytokine interferon-γ (IFN-γ), STAT1 becomes phosphorylated, dimerizes, and translocates to the nucleus, where it activates transcription of genes needed for resistance to pathogens. However, STAT1 can promote the transcription of different subsets of genes, depending upon the context in which its activation takes place. One explanation for this behavior could be that other cellular factors—for example, other transcription factors or DNA-binding proteins—can modulate the activity of STAT1 at different target genes. But if such factors exist, what are they? That’s the question Daniel Beiting, Sara Cherry, and colleagues sought to address in their paper published this month in *PLOS Biology* [1].

The researchers reasoned they might be able to find factors that modulate STAT1 activity by looking for proteins that can overcome STAT1 inhibition. Such inhibition frequently occurs during infection by certain pathogens, which attack STAT1 in order to evade detection and control by the immune system. The methods used by pathogens to frustrate STAT1 activity are as varied as the pathogens themselves; some manufacture proteins that interfere with STAT1 phosphorylation or nuclear translocation, whereas others specifically target STAT1 for degradation. Yet another strategy is the one used by the intracellular parasite *Toxoplasma gondii*, which allows STAT1 to reach the nucleus but then blocks the protein’s ability to activate its target genes in the nucleus. This pathogen likely attacks STAT1 because IFN-γ activity and STAT1 activity are essential for controlling *T*. *gondii* infections during both acute and systemic infections and are also important for restraining *T*.*gondii* replication in the brain, where the parasite encysts and persists during chronic infection (Fig 1).

**Fig 1:**
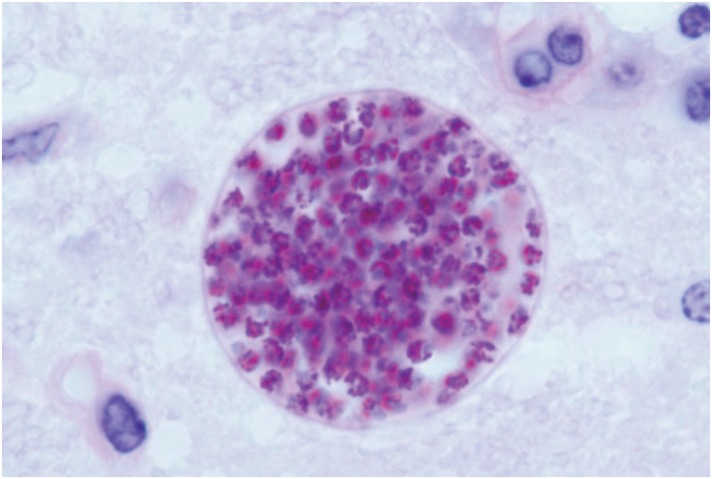
*T*. *gondii* cysts containing thousands of parasites (in red) can develop in many vertebrate tissues, such as the mouse brain. *Image credit: Jitender P. Dubey*.

Beiting and colleagues reasoned that there may be endogenous genes that, when ectopically expressed, can overcome the *T*. *gondii*–induced block on STAT1-mediated gene transcription. They therefore initiated a high-throughput screen, interrogating a library of 18,000 mouse and human cDNAs to identify genes that enhance STAT1 activity in *T*. *gondii*–infected cells. The screen turned up seven genes, each encoding transcription factors that specifically enhance STAT1 activity. Two of these, COUPTF2 and TLX, belong to the class of orphan nuclear receptors—transcriptional regulators whose natural ligands have not yet been identified but that often exhibit important functions in activating or repressing gene transcription. Because TLX is prominently expressed in the brain, the site of chronic *T*. *gondii* infections, the researchers decided to focus their efforts on understanding how this protein modulates STAT1 activity.

First, Beiting et al. sought to characterize the genes whose expression might be modulated by TLX by examining the effects of TLX overexpression in a human osteosarcoma cell line. They found that TLX by itself enhanced expression of more than 100 genes involved in neuron differentiation and tissue morphogenesis. However, in the presence of IFN-γ, overexpressed TLX also promoted the up-regulation of many genes related to immune function, including several that are known targets of STAT1. Conversely, knockdown of TLX in astroglioma cells, which express high levels of TLX endogenously, strongly attenuated transcription of a subset of IFN-γ-responsive genes, some of which are also targets of STAT1.

The authors next investigated what features of TLX are needed for potentiation of STAT1 targets and showed that both the protein’s DNA binding domain and its presumptive ligand binding domain were required to modulate STAT1-mediated transcription. Further, they found that TLX works by increasing the amount of phosphorylated STAT1 present at the promoter regions of certain STAT1 target genes. Together, these data suggest that TLX may modulate expression of a subset of STAT1 targets by binding to DNA and helping recruit STAT1 or increase its occupancy at particular targets to initiate transcription.

Finally, because TLX appears to be intimately involved in immune signaling by IFN-γ and STAT1, the authors investigated what happens to TLX during *T*. *gondii* infections. Strikingly, examination of brains of *T*. *gondii*–infected mice showed that TLX expression was strongly enhanced in many different cell types near encysted parasites. What’s more, TLX proved to be important for defense against *T*. *gondii*, as shown by the fact that the knockout of the protein in adult mice impaired the secretion of a cytokine important for T cell activation. Loss of TLX in the brain also increased parasite burden in chronically infected animals.

Together, these data demonstrate that TLX is a STAT1 transcriptional enhancer important for protection from *T*. *gondii* infection. Might TLX itself be targeted for attack by *T*. *gondii* or other pathogens? More work will be needed to find out, but in the meantime, this paper begins to explain how STAT1-dependent responses might be tailored in different cellular contexts.
